# Epidemiology and Risk Factors for Hearing Damage Among Adults Using Headphones via Mobile Applications

**DOI:** 10.7759/cureus.25532

**Published:** 2022-05-31

**Authors:** Arti Gupta, Satvinder S Bakshi, Rakesh Kakkar

**Affiliations:** 1 Department of Community and Family Medicine, All India Institute of Medical Sciences, Mangalagiri, Guntur, IND; 2 Department of ENT, All India Institute of Medical Sciences, Mangalagiri, Guntur, IND; 3 Department of Community and Family Medicine, All India Institute of Medical Sciences, Bathinda, Bathinda, IND

**Keywords:** hearwho, subclinical, preventable, headphone, audio, application, hearing loss

## Abstract

Background

Exposure to recreational noise is an emerging public health problem leading to hearing loss. Young people often spend their leisure and relaxation time listening to unsafe levels of music on their personal audio systems. This study aimed to study hearing impairment among young adults due to exposure to various audio gadgets.

Methodology

This cross-sectional study was conducted among 241 patients and healthcare workers aged 20-40 years. The data collection instrument was a questionnaire including the history and patterns of usage of headphones. A mobile application-based hearing test, a validated Smartphone hearing screening technology, by the World Health Organization (hearWHO) was used to check hearing.

Results

Of the 241 adult participants (aged 20 to 40 years), 188 (78%) were males and 53 (22%) were females. The mean hearWHO score was 46.02 ± 9.854, ranging from 21 to 85. The mean hearWHO score for headphone usage with a music system of 50.45 ± 12.32 (p = 0.023) and television of 44.13 ± 8.595 (p = 0.015) was statistically significant. The mean hearWHO score for daily headphone users was 45.93 ± 9.67, for users using daily for more than two hours was 46.59 ± 10.34, for those using headphones for leisure was 47.51 ± 7.74, and for usage music/gaming was 48.71 ± 12.57. Subclinical hearing loss was seen in 201 (83.4%) headphone users. A higher proportion of subclinical hearing loss (74.1%) was noted among participants who used headphones for multiple reasons such as leisure, education, service, music, and gaming.

Conclusions

In this study, a high proportion of headphone users were found to have subclinical hearing loss. This study generated imperative facts for people and emphasize that they look after their hearing. This study introduces the applicability of new technology in an Indian setting where hearing healthcare is facing challenges.

## Introduction

Exposure to recreational noise is an emerging public health problem leading to hearing loss. Young people often spend their leisure and relaxation time listening to unsafe levels of music on their personal audio systems or are exposed to high levels of music or noise at concerts, bars, sports arenas, and clubs [1]. The highest safe exposure level up to a maximum of eight hours is 85 dB. The permissible time for safe listening decreases as sound levels increase [2].

According to the National Health and Nutrition Examination Survey conducted in the United States in 2005, the prevalence of hearing loss among 12-19-year olds is 5.3%. It is estimated to be proportional to an increased number of people listening to music through headphones. A 2008 European Commission report documented an increase in the use of personal audio devices by the population [2]. The World Health Organization (WHO) estimated the prevalence of auditory impairment in 6.3% of the Indian population based on the National Sample Survey Office (NSSO) 2001 data [3]. The increasing technological development is a potential risk for hearing loss. Increased accessibility and use of personal audio devices are associated with their use at high volumes and for long durations [2]. Such behaviors are associated with the risk of permanently damaging hearing capacity in the new techno-savvy generation [2]. Young adults are using mobile phones excessively not only for music, online transactions, and applications but also for educational purposes [4].

Some of the other harmful effects of listening through these hearing gadgets include ear infection, earache, numbness, adverse effect on the brain, congested air passage, external threats, hyperacusis, and multiple sclerosis. Over usage of headphones also increases psychological distress leading to becoming less attentive, unsocial, ignorant, less active, and thereby affecting individuals’ potential. Excessive usage of hearing gadgets may also lead to external threats such as accidents and even loss of life [5,6].

Considering the seriousness of the newly arising challenge of new technologies and the cumulative effects, it is essential to study both qualitatively and quantitatively the impact of these gadgets on health if used excessively. This study aimed to investigate hearing impairment among young adults due to exposure to various audio gadgets.

## Materials and methods

This cross-sectional study was conducted in the Departments of Community and Family Medicine and ENT, All India Institute of Medical Sciences (AIIMS), Mangalagiri, Andhra Pradesh, from October 2020 to March 2021. Male and female patients and healthcare workers aged 20-40 years were enrolled in the study. The possibility of sensorineural deafness increases above the age of 40. In this study, we included employees or patients of AIIMS, Mangalagiri using headphones. The study participants needed to understand spoken numbers between 1 and 10. Patients who were very sick; had a history of ear surgery, history of diabetes, deafness, ear discharge, or any ear disease; were on ototoxic drugs; and were living/working in a noisy environment were excluded from the study. The sample size was calculated based on the prevalence of subclinical hearing loss of 15.6% in a study of the epidemiology and risk factors for leisure noise-induced hearing among Flemish young adults [7]. The sample size was calculated to be 210 at 5% precision and 95% confidence level. Assuming a non-response of 15%, the final sample size was 241.

The data collection instrument was a questionnaire including information on patient demographics, namely, age, sex, marital status, residence, and others. The questionnaire included questions regarding subjective hearing status and medical history concerning ear block tinnitus, sound sensitivity, etc. In addition, questions on the history of usage of headphones, the duration of use, the maximum volume, whether participants slept with audio gadgets on play, and a history of other health problems were also included [8]. We utilized a mobile application-based hearing test using a validated smartphone hearing screening technology by the WHO (hearWHO). hearWHO is a mobile application that conducts hearing screening. The screening analyzes the person’s ability to perceive speech-in-noise. Based on the person’s response, the app automatically generates a hearing score that indicates whether there may be a possible hearing loss. It is a mobile application that conducts hearing screening. The screening analyzes the person’s ability to perceive speech-in-noise by presenting 23 sets of US English digit triplets over ever-increasing white noise. Based on the person’s response, the app automatically generates a hearing score that indicates whether there may be a possible hearing loss. If the person fails, the app indicates the need to visit an audiologist. The hearWHO app is based on validated digits-in-noise technology. A score of above 75 signifies good hearing. A score of 50 and 75 require hearing check regularly to know if the score reduces further. A score of <50 signifies some degree of hearing loss and the need to visit an audiologist. The app is presently available in English only [9].

Patients attending routine outpatient units (OPDs) and eligible employees were randomly sampled and enrolled in the study. Investigators introduced themselves to study participants before the start of the interview. Study participants were given an information sheet and were explained the study objectives, procedure, and the rights of the participants. If the study participant agreed to participate in the study after going through the information sheet, written consent was taken from him/her. A video in English/Telugu was played to describe the study procedure. The study participants were interviewed according to the questionnaire in a separate barrier area in the OPD of the Department of Community and Family Medicine. All measures to prevent any risk of contracting coronavirus disease 2019 were strictly adhered to, including handwashing, mask usage, and social distancing, among others. Subsequently, the mobile application-based hearing test was conducted following the standard procedure. If participants had access to the mobile application, they were motivated to use their phones. For others, disposable headphones were used, and tablets were covered with polythene films. Any biomedical waste generated was disposed of as per the guidelines. The survey was continued until the final sample size was achieved. Any study participants provisionally diagnosed with hearing loss were referred to ENT for further evaluation.

Operationally, study participants with hearing loss were defined as participants’ self-reported hearing loss, or as reported by the mobile-based hearing screening test with a score less than 50 [9]. The dependent variable was hearing loss, and the independent variables included demographic variables and the history of usage of headphones. All data were entered into Microsoft Excel 2010. Statistical analysis using descriptive and inferential methods was performed using SPSS version 17 software (SPSS Inc., Chicago, IL, USA). Graphs were produced using Microsoft Excel software after obtaining the relevant information from the SPSS output. Descriptive methods were used to obtain frequency tables and graphs. Inferential methods were used for the association between selected socio-demographic variables, audio gadget use, attitude toward noise or other variables, and hearing loss. Logistic regression was used to identify the predictors of hearing loss. All hypotheses were tested at a 5% level of significance. Ethical clearance was received from the Institutional Ethics committee, All India Institute of Medical Sciences, Mangalagiri, Andhra Pradesh, India (AIIMS/MG/IEC 2020-21-50).

## Results

**Figure 1:**
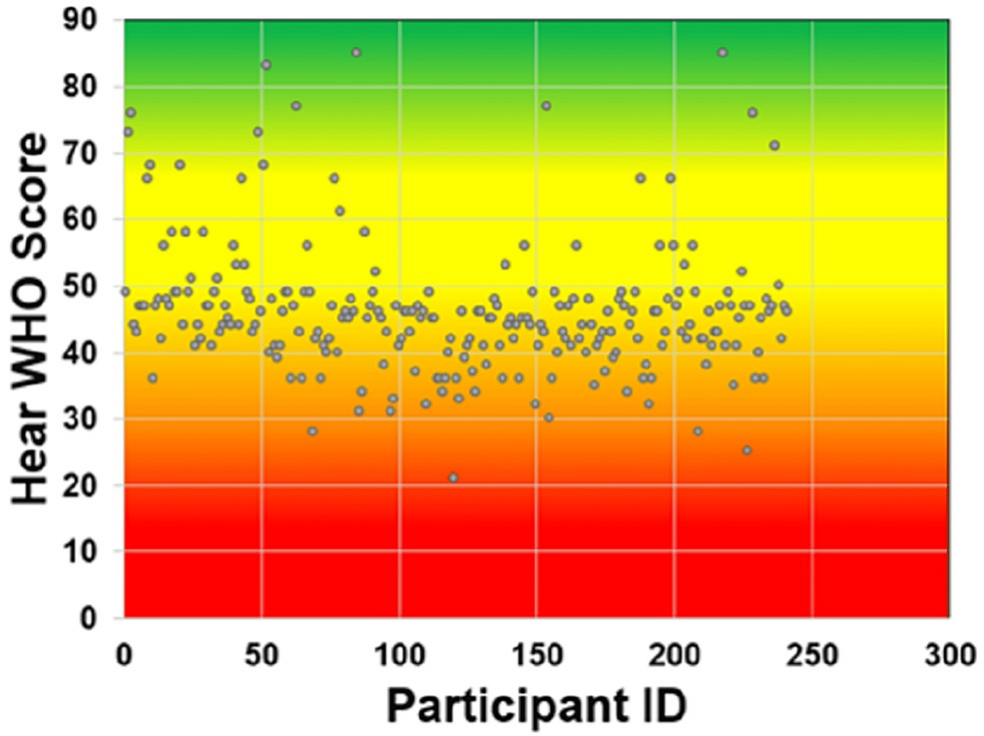
hearWHO scores among the study participants.

Data analysis was conducted on the findings of 241 adults (20 to 40 years). In total, 188 (78%) males and 53 (22%) females participated in the study (Table 1). The mean hearWHO score was 46.02 ± 9.854, ranging from 21 to 85 (Figure 1).

**Table 1:** Distribution of hearWHO score by sociodemographic factors and clinical features (n = 241). SD: standard deviation

Variable	Category	hearWHO score		
		Mean	SD	P-value
Age (years)	≤20	45.63	8.03	0.004
	21–35	47.3	10.2	
	≥36	42.13	8.49	
Gender	Male	45.92	9.34	0.776
	Female	46.36	11.56	
Occupation	Office job	46.81	10.396	0.23
	Not working	44.30	4.945	
	Student	45.86	8.731	
	Daily wage workers	43.48	8.772	
Education	Higher secondary	45.45	10.083	0.035
	Secondary	46.68	8.903	
	Graduate	47.70	10.411	
	Postgraduate	45.29	8.824	
	Not literate	38.00	5.865	
Chronic illness	Yes	43.25	10.490	0.246
	No	46.21	9.800	
Ask people to repeat what was said	Yes	47.03	9.760	0.533
	No	45.86	9.880	
Increase television volume	Yes	45.25	8.400	0.425
	No	46.35	10.429	
Sleep disturbance	Yes	43.74	7.62	0.14
	No	46.4	10.15	
History of raised blood pressure in the last three months	Yes	44.43	9.970	0.536
	No	46.11	9.860	
Restless without audio gadget/headphone	Yes	47.5	11.7	0.594
	No	45.94	9.77	

**Figure 2:**
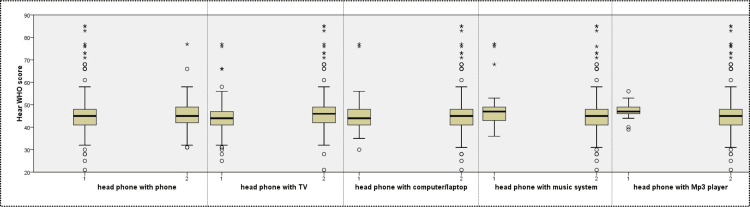
Box plot showing hearWHO score with devices (1 is yes, 2 is no). *: extreme outliers; O: mild outliers

Headphone use with computers/laptops, televisions (TVs), phones, music systems, Mp3 players, and others was 21.2%, 40.2%, 84.2%, 9.1%, 5.4%, and 3.7%, respectively. The mean hearWHO score for headphone users with a music system was 50.45 ± 12.32 (p = 0.023) and for headphone users with TV was 44.13 ± 8.595 (p = 0.015); this finding was statistically significant (Figure 2).

The mean hearWHO score for daily headphone users was 45.93 ± 9.67, for users using daily for more than two hours was 46.59 ± 10.34, for users using headphones for leisure was 47.51 ± 7.74, and for those using headphones for music/gaming was 48.71 ± 12.57 (Table 2).

**Table 2:** Distribution of hearWHO scores by the patterns and usage of headphones (n = 241). SD: standard deviation

Variable	Category	hearWHO score		
		Mean	SD	P-value
Frequency of headphone use	Daily	45.98	9.673	0.999
	Alternate Day	46.04	10.609	
	Weekly once or twice	46.07	7.292	
Duration of headphone use	Less than 1 hour	45.63	10.796	0.827
	1–2 hours	46.14	7.785	
	More than 2 hours	46.59	10.343	
Purpose to use headphones	Leisure	47.51	7.740	0.282
	Education/Service	42.62	6.888	
	Music/Gaming	48.71	12.579	
	More than one option/Other	46.02	10.360	
Maximum volume used (out of 10 points)	<5	44.99	11.125	0.298
	5–7	46.05	8.468	
	>7	47.90	10.797	
Asked to reduce the volume of headphones	Yes	45.58	7.890	0.783
	No	46.09	10.140	
Drive a vehicle with loud music	Yes	49.3	11.35	0.039
	No	45.5	9.52	
Restless without audio gadget/headphone	Yes	47.5	11.7	0.594
	No	45.94	9.77	
Sleep with headphones on play	Yes	46.74	8.29	0.712
	No	45.94	10.018	

Subclinical hearing loss with hearWHO scores of less than 50 was present among 201 (83.4%) headphone users. Nearly 66.2% of participants in the age group of 21-35 years and 79.1% of males had subclinical hearing loss (Table 3). A higher proportion of subclinical hearing loss (74.1%) was noted among participants who used headphones for multiple reasons such as leisure, education, service, music, and gaming (Table 4).

**Table 3:** Distribution of subclinical hearing loss by sociodemographic factors and clinical features (n = 241). SD: standard deviation

Variable	Category	Subclinical hearing loss (201)		Normal (40)		Chi square	P-value
		n	%	n	%		
Age (years)	≤20	20	10.0%	4	10.0%	3.911	0.141
	21–35	133	66.2%	32	80.0%		
	≥36	48	23.9%	4	10.0%		
Gender	Male	159	79.1%	29	72.5%	0.848	0.357
	Female	42	20.9%	11	27.5%		
Occupation	Office job	136	67.7%	30	75.0%	1.537	0.674
	Not working	9	4.5%	1	2.5%		
	Student	17	8.5%	4	10.0%		
	Daily wage workers	39	19.4%	5	12.5%		
Education	Higher secondary	68	33.8%	12	30.0%	8.476	0.076
	Secondary	27	13.4%	7	17.5%		
	Graduate	62	30.8%	19	47.5%		
	Postgraduate	33	16.4%	2	5.0%		
	Not literate	11	5.5%	0	0.0%		
Chronic illness	Yes	15	7.5%	1	2.5%	1.326	0.250
	No	186	92.5%	39	97.5%		
Ask people to repeat what was said	Yes	27	13.4%	5	12.5%	0.025	0.874
	No	174	86.6%	35	87.5%		
Increase television volume	Yes	63	31.3%	10	25.0%	0.636	0.425
	No	138	68.7%	30	75.0%		
Sleep disturbance	Yes	32	15.9%	3	7.5%	1.905	0.167
	No	169	84.1%	37	92.5%		
History of raised blood pressure in the last three months	Yes	13	6.5%	1	2.5%	0.960	0.327
	No	188	93.5%	39	97.5%		
Restless without audio gadget/headphone	Yes	10	5.0%	2	5.0%	0.000	0.995
	No	191	95.0%	38	95.0%		

**Table 4:** Distribution of hearWHO score and subclinical hearing loss by the patterns and usage of headphones (n = 241). *Significant with p-values of <0.05.

Variable	Category	Subclinical hearing loss (201)		Normal (40)		Chi square	P-value
		n	%	n	%		
Frequency of headphone use	Daily	85	42.3%	15	37.5%	3.144	0.208
	Alternate day	90	44.8%	23	57.5%		
	Weekly once or twice	26	12.9%	2	5.0%		
Duration of headphone use	Less than 1 hour	93	46.3%	18	45.0%	0.253	0.881
	1–2 hours	60	29.9%	11	27.5%		
	More than two hours	48	23.9%	11	27.5%		
Purpose to use headphones	Leisure	27	13.4%	8	20.0%	1.789	0.617
	Education/Service	19	9.5%	2	5.0%		
	Music/Gaming	6	3.0%	1	2.5%		
	More than one option/Other	149	74.1%	29	72.5%		
Maximum volume used (out of 10 points)	<5	67	33.3%	14	35.0%	0.049	0.976
	5–7	99	49.3%	19	47.5%		
	>7	35	17.4%	7	17.5%		
Asked to reduce the volume of headphone	Yes	29	14.4%	4	10.0%	0.553	0.457
	No	172	85.6%	36	90.0%		
Drive a vehicle with loud music	Yes	23	11.4%	10	25.0%	5.188	0.023*
	No	178	88.6%	30	75.0%		
Restless without audio gadget/headphone	Yes	10	5.0%	2	5.0%	0.000	0.995
	No	191	95.0%	38	95.0%		
Sleep with headphone on play	Yes	19	9.5%	4	10.0%	0.012	0.914
	No	182	90.5%	36	90.0%		

In the hierarchical stepwise multiple linear regression analysis, the overall hearWHO score had a mean of 4.05 ± 0.45. Demographic factors (Block 1, Table 5) explained 1.5% of the variance (adjusted R^2^ = 0.150) in the overall hearWHO score when none of the other factors was controlled for. The socioeconomic factors and patterns of usage of headphone variables (Block 2 and 3, Table 5) explained an additional 5% and 12% of the variance. The clinical features added 2% to the hearWHO score variance. Altogether, the final model explained 34% of the variance of hearWHO scores (Table 5).

**Table 5:** Results of hierarchical stepwise multiple linear regression analyses. *Significant with p-values of <0.05.

Variable	R	Category	B	95% confidence interval for B		Beta	P-value
				Lower bound	Upper bound		
Block 1							
Demographic factors	0.150	Age	-1.671	-4.358	1.016	-0.093	0.22
		Gender	1.452	-1.851	4.755	0.061	0.39
Block 2							
Social factors	0.208	Occupation	-0.823	-1.996	0.350	-0.101	0.17
		Education	-0.532	-1.652	0.589	-0.066	0.35
Block 3							
Pattern of headphone usage	0.328	Headphone use with computer/laptop	1.009	-2.746	4.764	0.042	0.60
		Headphone use with phone	0.606	-3.181	4.393	0.022	0.75
		Headphone use with music system	-6.253	-10.985	-1.522	-0.183	0.01
		Headphone use with Mp3 player	2.548	-3.689	8.786	0.059	0.42
		Headphone use with television	1.969	-1.151	5.090	0.098	0.21
		Frequency of headphone use	0.100	-2.087	2.287	0.007	0.93
		Duration of headphone use	0.518	-1.388	2.423	0.043	0.59
		Purpose to use headphone	0.179	-1.021	1.378	0.021	0.77
		Maximum volume of headphone use (out of 10 points)	1.438	-0.607	3.484	0.102	0.17
		Asked to reduce the volume of headphone	2.341	-1.597	6.279	0.082	0.24
		Sleep with headphone on play	-.432	-4.846	3.983	-0.013	0.85
Block 4							
Clinical features	0.344	Chronic illness	2.703	-2.661	8.067	0.068	0.32
		Raised blood pressure in the last three months	-1.906	-7.651	3.839	-0.045	0.51
		Restless without audio gadget/headphone	1.136	-5.238	7.510	0.025	0.73
		Sleep disturbance	2.164	-1.751	6.078	0.078	0.28
		Need to increase television volume	1.516	-1.506	4.537	0.071	0.32
		Need to ask people to repeat what was said	-0.559	-4.529	3.412	-0.019	0.78
		Drive a vehicle with loud music	-4.024	-8.048	-0.001	-0.141	0.05

## Discussion

Hearing damage is linked to problems with day-to-day activities. Hearing loss can restrict social interactions, decrease quality of life, and cause psychological issues, such as feelings of loneliness and alienation, depression, and cognitive disabilities in children and adults [10]. Hearing loss is at least partly linked to short-term or prolonged exposure to loud sounds in almost half of the people who suffer from it [11].

Noise exposure is a significant cause of hearing loss. Many people, even those who listen to music on personal music devices, are subject to noise willingly, although it may be unintentional. For factory employees who are subjected to an equivalent sound frequency of >85 dB of noise for eight hours a day, ear safety caps or noise reduction are advised. However, the average listener’s volume is set between 75 and 100 dB [12], and people who listen to 15 minutes of music at 100 dB on personal music players can be subject to the same amount of noise as factory employees who are exposed to 85 dB over an eight-hour day [13]. Consequently, noise-induced hearing loss can be harmful.

In developing nations like Africa, where the burden of disabling hearing loss is the highest, access to ear and hearing protection was a problem [14]. This study examined using mobile hearing screening (hearWHO) to detect hearing damage in people using headphones. This study examined the hearing abilities of 241 young Indian people between the ages of 20 and 40. For the majority of the participants, the hearWHO scores were below 50, the cut-off for standard listening ranges. Similarly, studies have reported that a significant number of young people suffer from hearing deprivation because of noise exposure [15-17]. On the other hand, other surveys have shown no substantial hearing loss in young adults in their research [18,19]. There are some methodological variations between experiments, such as the operational definition of a normal-hearing person and the validity and reliability of the hearing test used, which can contribute to contradictory findings. In this regard, the current analysis used a strict set of inclusion parameters, and hearing acuity was determined using hearWHO, an android application.

Gender was not statistically correlated with hearing status in both univariate and multivariate analyses. In the univariate analysis, however, age was shown to be strongly associated with subclinical hearing loss. However, we could not predict clinical age-related hearing improvements in such a young age group based on the well-known relationship between age and hearing deterioration [20]. According to the multivariate analysis, however, age was not related to an elevated risk of subclinical hearing loss. Similarly, another study examined community hearing loss detection using smartphone hearing screening (hearScreen^TM^) by community health professionals and discovered that adult referral rates were greatly impaired by age (p = 0.05) [21]. Furthermore, illiteracy and low educational status were found to be univariately correlated with hearing status, with working participants having more subclinical hearing loss than college graduates. According to the multivariate analysis, however, literacy status was not associated with an increased risk of subclinical hearing loss. This can be explained by the fact that multivariate tests take into account interdependencies between variables.

Excessive noise exposure has been identified as a risk factor for hearing loss in several studies [22,23]. The current survey found that the majority of young adults in this study used headphones for various activities, with varying levels of duration and noise levels for each day. In accordance with previous research, a higher proportion of subclinical hearing loss was present among our study participants who used headphones for multiple reasons such as leisure, education, service, music, and gaming. However, no significant relationships between hearing status and noise exposure were observed, which is consistent with previous studies [24,25]. According to a survey of Flemish young adults aged 18 to 30, no consistent associations between a participant’s actual hearing status and recreational noise exposure were found [7]. One potential reason for this finding is that the duration of use of headphones, and therefore the rate of noise exposure, did not vary widely enough among study participants. Subjectively, headphone usage and sound exposure were noted in this study but did not display significantly poorer assessed hearing thresholds. One reason for this finding may be that the key problem is not the history of using headphones but rather how and where it is used, such as volume, exposure time, and sound setting, among others [10].

Hearing screening on a smartphone enables primary and secondary healthcare doctors to provide hearing care to underserved populations. Quality control and remote tracking for surveillance and follow-up are possible owing to active noise monitoring and data collection functionality. Studies have shown show that the computerized self-administered hearing test is a legitimate, accurate, and cost-effective method of determining unmasked air-conduction hearing thresholds [4-7]. Because of the current growth of personal music players and cell phones, exposure to loud music has risen dramatically [26]. The gold standard for hearing screening services is pure-tone screening. Audiometric screening should be performed on handheld devices such as mobiles and tablets facilitating early detection of hearing loss. On World Hearing Day in March 2016, the hearZA app, South Africa’s national hearing test, was unveiled [14]. In underserved areas, using the hearing application test at small health settings and primary health centers can reduce the demand for audiological services.

There are some limitations of this study. First, measuring sound exposure, that is, self-reported time, frequency, and sound levels of usage may be inaccurate, and no real loudness measures were taken to quantify the A-weighted equivalent levels. Second, both tonal audiometry and otoacoustic emissions were not used to identify participants with clinical hearing damage in this study. Furthermore, because the educational qualifications of the participants varied, their understanding may differ regarding the subjective evaluation of noise, and people may be biased to report their hearing status.

## Conclusions

Hearing is precious, and hearing damage due to excessive noise is irreversible. For affected people, quality of life deteriorates and healthcare costs can increase. Noise-induced hearing loss is preventable. In this study, a high proportion of headphone users were found to have subclinical hearing loss using the hearWHO application at the hospital level. This study generated imperative facts for people and emphasize the need to look after their hearing. This advocates for institution and program managers to develop standards to limit exposure to recreational noise. Furthermore, hearing-related services are often overlooked at the primary healthcare level. This study introduces the applicability of the new technology in an Indian setting where hearing healthcare is facing challenges. In developing countries with low access to hearing healthcare at the facility level, the hearWHO screen guides primary healthcare-level doctors to identify patients requiring a referral to an ENT specialist.
